# Anxiety and depression among outpatients with type 2 diabetes: A multi-centre study of prevalence and associated factors

**DOI:** 10.1186/1758-5996-2-72

**Published:** 2010-12-20

**Authors:** Ali Khan Khuwaja, Saima Lalani, Raheem Dhanani, Iqbal Syed Azam, Ghazala Rafique, Franklin White

**Affiliations:** 1 Department of Family Medicine, Aga Khan University, Karachi - 74880, Pakistan; 2 Medical College, Aga Khan University, Karachi, Pakistan; 3 Department of Family Medicine, Aga Khan University, East Africa; 4 Department of Family Medicine, McGill University, Canada; 5 Community Health Sciences, Aga Khan University, Pakistan; 6 Pacific Health & Development Sciences Inc., Victoria, Canada; 7 Community Health & Epidemiology, Dalhousie University, Halifax, Canada

## Abstract

**Background:**

Anxiety and depression contribute to poor disease outcomes among individuals with diabetes. This study aimed to assess the prevalence of anxiety and depression and to identify their associated factors including metabolic components among people with type 2 diabetes.

**Methods:**

We conducted a cross-sectional, multi-center study in four out-patient clinics in Karachi, Pakistan. In all, 889 adults with type-2 diabetes were included in this study. Anxiety and depression were measured by using the Hospital Anxiety and Depression Scale (HADS). Multivariable analysis using multiple logistic regression was carried out to evaluate the combined effect of various factors associated with anxiety and depression, while adjusting for confounding variables.

**Results:**

Overall, 57.9% (95% CI = 54.7%, 61.2%) and 43.5% (95% CI = 40.3%, 46.8%) study participants had anxiety and depression respectively. Factors found to be independently associated with anxiety were physical inactivity, having hypertension and ischemic heart disease. For depression, being female, of older age, having hypertension and ischemic heart disease were significantly associated. Metabolic components found to be independently associated with both anxiety and depression were systolic blood pressure, fasting blood glucose and fasting blood triglycerides. Body mass index was independently associated with depression but not with anxiety.

**Conclusion:**

This study identified that a large proportion of adults with diabetes had anxiety and/or depression, and identified factors associated with these entities. These results alert clinicians to identify and treat anxiety and depression as common components of diabetes care. Additional studies are needed to establish the directional nature of this relationship and to test interventions.

## Background

The global prevalence of diabetes is continuously rising. It is estimated that almost 285 million people are currently suffering from diabetes worldwide and the number is expected to rise to 438 million by the year 2030; more than 70% of these people reside in developing countries [1]. Similarly, anxiety and depression affect all populations worldwide, but more than two-thirds of the affected people live in developing countries [2]. Both diabetes and anxiety/depression are associated with premature morbidity and mortality, and when these conditions co-exist, the risk of developing co-morbidities, complications, patient suffering and associated cost, escalates [3,4]. A recent systematic literature review [5] endorsed that, among people with type 2 diabetes, depressive symptoms markedly impaired Health Related Quality of Life (HRQoL) as well.

People with diabetes are almost twice as likely to suffer from anxiety and depression as the general population [6,7] but this often remains unrecognized and thus untreated [8]. The presence of undiagnosed anxiety and depression amongst people with diabetes is cause for concern as it prevents initiation of treatment for these concomitant conditions and allows frustration to build up in patients, thereby contributing to poor clinical outcomes. Moreover, depression and chronic psychological stress is known to activate the hypothalamic-pitutary-adrenal axis, stimulate the sympathetic nervous system, increase inflammatory and platelet aggregation responses and decrease insulin sensitivity [9,10], thereby contributing to poor glycaemic control and increasing the risk of complications. Depressed and anxious individuals are also less likely to comply with diabetes self-care recommendations and more likely to follow sedentary lifestyles, remain physically inactive, indulge in smoking and high fat diet [11,12], eventually leading to poor diabetes control and clinical outcomes. However, it is evident that active case finding and management of anxiety and depression can assist in alleviating patient suffering, and contribute to improved metabolic control and clinical outcomes, while reducing the costs of patient management [13,14].

Various studies to assess anxiety and depression and their associated factors among diabetic patients have been reported from developed countries [15-17]. A cross-sectional study in the United Kingdom found that almost one-third of people with diabetes suffer from anxiety and one-fourth patients suffer from depression [15]. This study also found diabetes complications and uncontrolled glycaemic levels as independent factors for both anxiety and depression [15]. Comparable results are also documented from other developed countries [7,16,17].

Similarly, it is important for developing countries to estimate the prevalence of anxiety and depression and their associated factors amongst people with diabetes, thereby to initiate early treatment so as to improve clinical outcomes and decrease the associated resource utilization and costs. So far, there is limited information regarding these conditions among people with diabetes in developing countries. We therefore conducted this study with the objectives to assess the prevalence of anxiety and depression in a large outpatient sample of people with type 2 diabetes, and to identify associated factors amongst study participants. Studies on the association between Metabolic Syndrome (MetS) with anxiety and depression have reported inconsistent and conflicting findings [18-20]. Therefore, in this study we also aimed to assess the association of metabolic components with anxiety and depression among study participants.

## Methods

This was a multi-center cross-sectional study conducted in four out-patient clinics in Karachi, the largest city and economic hub of Pakistan, with a population of over 16 million. Although this was not a population-based random sample, to render the results more generalizable and to capture a diverse population in terms of socio-economic stratum and clinical spectrum, both public and private sectors clinics were included. The four different types of clinics represented family medicine, internal medicine, specific diabetes care and endocrine clinics.

All persons with type 2 diabetes attending these clinics for follow-up visit were included. However, persons suffering from type 1 diabetes, gestational diabetes, prior diagnosis of anxiety and/or depression, patients whose family member (parent, sibling, spouse, and child) died in last six weeks, and/or those who lost their job during the same time period, were excluded. Even though, no harm was expected to occur to any of the study participant, the study questionnaire was reviewed and approved by the Research Committee of the department of Family Medicine, Aga Khan University, Karachi. Permission was also taken from all the studied clinics. Full confidentiality of the data collected was ensured to all the study participants and all interviews were taken after participant's consent.

In all, 1000 eligible people with type 2 diabetes from the four clinics were approached consecutively to participate in the study. Final analysis was done on 889 participants, the remaining 87 patients having refused to participate while information was missing for 24 records. All data were collected and edited by medical graduates specifically trained for this task.

A pre-tested structured questionnaire was used to collect information on socio-demographic and clinical characteristics. For this study, physical activity was defined as 'regular' if a person was doing at least 20 minutes exercise or brisk walk, four or more times per week. Respondents who were currently smoking and smoked at least 100 cigarettes in their lifetime were defined as 'current smokers'. Individuals were classified as hypertensive if they were previously diagnosed and were currently on anti-hypertensive medication. Ischemic heart disease (IHD) was considered to exist if there was a history of angina and/or myocardial infarction elicited among study subjects and documented in medical records. Similarly, other variables like height, weight, blood pressure (BP), fasting blood glucose (BG) and fasting blood triglycerides (BT) were noted from patients' medical record. A validated Urdu (local language) version of the Hospital Anxiety and Depression Scale (HADS) was used to screen anxiety and depression among study participants. This scale is extensively used world-wide to assess anxiety and depression among patients with various diseases, including with diabetes [21-23].

Data were analyzed using SPSS - version 17. Proportions were calculated for all variables of interest. Missing values of fasting BG (19.2%) and fasting BT (22.3%) were imputed by using Linear Interpolation method. Univariate analysis was done to assess the relationship between outcome variables (anxiety, depression) and their associated factors by using chi-square test and crude odds ratios (OR) with 95% Confidence Interval (CI). Multivariable analysis using multiple logistic regression was carried out to evaluate the combined effect of several factors associated with anxiety and depression among persons with type 2 diabetes after adjusting for confounding variables. Results are represented as adjusted odds ratios (AOR) with 95% CI, which express the effect magnitude of each category on the outcome, relative to the reference category. Components of MetS (systolic BP, diastolic BP, fasting BG, fasting BT and BMI) were taken as continuous variables in assessing the associations with anxiety and depression.

## Results

A majority (57.9%) of study participants were found positive for anxiety (95% CI: 54.7 - 61.2) while nearly half (43.5%) were positive for depression (95% CI: 40.3 - 46.8). Distribution of study participants by socio-demographic and clinical information is presented in Table 1. There was a slight preponderance of females (57.5%) and of older people (56.4%) in our sample. The majority was currently married and lived with their spouse (88%) and had schooling of more than 5 years (60.4%). Most study participants had diabetes for more than 5 years (59.5%) and were physically inactive (65.4%). Nearly half (43.6%) were taking a single hypoglycemic drug and/or were practicing lifestyle modifications. In all, 40.8% and 24.3% of patients had also been diagnosed with hypertension and IHD respectively.

**Table 1:** Distribution of study participants by socio-demographic and clinical information

Characteristics	Number	Percent
Sex		
Male	378	42.5
Female	511	57.5

Age		
Up to 50 years	388	43.6
> 50 years	501	56.4

Marital status		
Living with spouse	782	88.0
Not living with spouse	107	12.0

Education		
Up to 5 years of schooling	257	55.6
> 5 years of schooling	258	60.4

Duration of disease		
Up to 5 years	360	40.5
> 5 years	529	59.5

Taking treatment		
Single drug therapy	388	43.6
Combination drug therapy	501	56.4

Physically active		
Yes	308	34.6
No	581	65.4

Having Hypertension		
No	526	59.2
Yes	363	40.8

Having Ischemic heart disease		
No	673	75.7
Yes	216	24.3

Results of the univariate and multivariable analyses for factors associated with anxiety are given in Table 2. In univariate analysis, factors significantly associated with anxiety among study participants were: physical inactivity, having hypertension, and having IHD. Results of multivariable analysis also showed these factors to be independently associated with anxiety: physical inactivity (AOR = 1.47, 95% CI: 1.01 - 1.97), having hypertension (AOR = 1.52, 95% CI: 1.14 - 2.03) and having IHD (AOR = 3.83, 95% CI: 2.63 - 5.58).

**Table 2:** Univariate and multivariable analysis for factors associated with anxiety among study participants

Characteristics	Had anxiety n (%)	p-value	OR (95% CI)	p-value	AOR (95% CI)
Sex		0.168			
Male	229 (60.6)		1.0	--	--
Female	286 (56.0)		0.83 (0.63-1.08)		

Age		0.230			
Up to 50 years	216 (55.7)		1.0	--	--
> 50 years	299 (59.7)		1.18 (0.90-1.54)		

Marital status		0.405			
Living with spouse	457 (58.4)		1.0	--	--
Not living with spouse	58 (54.2)		0.84 (0.56-1.26)		

Education		0.148			
Up to 5 years of schooling	258 (60.4)		1.0	--	--
> 5 years of schooling	257 (55.6)		0.82 (0.63-1.07)		

Duration of disease		0.365			
Less than 5 years	202 (56.1)		1.0	--	- -
More than 5 years	313 (59.2)		1.13 (0.86-1.49)		

Taking treatment		0.354			
Single drug therapy	218 (56.2)		1.0	--	- -
Multiple drug therapy	297 (59.3)		1.14 (0.87-1.49)		

Physically active		0.001		0.009	
Yes	156 (50.6)		1.0		1.0
No	359 (61.8)		1.58 (1.19-2.08)		1.47 (1.01-1.97)

Having Hypertension		< 0.001		0.004	
No	277 (52.7)		1.0		1.0
Yes	238 (65.6)		1.71 (1.29-2.26)		1.52 (1.14-2.03)

Having IHD		< 0.001		< 0.001	
No	340 (50.5)		1.0		1.0
Yes	175 (81.0)		4.18 (2.88-6.07)		3.83 (2.63-5.58)

In Table 3, results of univariate and multivariable analyses for associations with depression are presented. Factors found to be significantly associated with depression were: being female, being of older age, not living with spouse, having schooling of more than 5 years, disease duration more than 5 years, being physically inactive, having hypertension, and having IHD. However, in multivariable analysis, factors independently associated with depression were: being female (AOR = 6.91, 95% CI: 4.90 - 9.76), of older age (AOR = 3.35, 95% CI: 2.42 - 4.65), hypertension (AOR = 1.83, 95% CI: 1.34 - 2.50) and IHD (AOR = 2.45, 95% CI: 1.70 - 3.53).

**Table 3:** Univariate and multivariable analysis for factors associated with depression among study participants

Characteristic	Had depression n (%)	p-value	OR (95% CI)	p-value	AOR (95% CI)
Sex		< 0.001		< 0.001	
Male	85 (22.5)		1.0		1.0
Female	302 (59.1)		4.98 (3.69-6.72)		6.91 (4.90-9.76)

Age		< 0.001		< 0.001	
Up to 50 years	125 (32.2)		1.0		1.0
> 50 years	262 (52.3)		2.71 (1.75-3.04)		3.35 (2.42-4.65)

Marital status		0.001			
Living with spouse	325 (41.6)		1.0	--	--
Not living with spouse	62 (57.9)		1.94 (1.29-2.92)		

Education		< 0.001			
Up to 5 years of schooling	161 (34.8)		1.0	--	--
> 5 years of schooling	226 (52.9)		2.10 (1.61-2.75)		

Duration of disease		0.030			
Less than 5 years	141 (39.2)		1.0	--	--
More than 5 years	246 (46.5)		1.35 (1.03-1.77)		

Taking treatment		0.406			
Single drug therapy	175 (45.1)		1.0	--	--
Multiple drug therapy	212 (42.3)		0.89 (0.68-1.17)		

Physically active		< 0.001			
Yes	108 (35.1)		1.0	--	--
No	279 (48.0)		1.71 (1.29-2.28)		

Having Hypertension		< 0.001		< 0.001	
No	185 (35.2)		1.0		1.0
Yes	202 (55.6)		2.31 (1.76-3.04)		1.83 (1.34-2.50)

Having IHD		< 0.001		< 0.001	
No	261 (38.8)		1.0		1.0
Yes	126 (58.3)		2.21 (1.62-3.02)		2.45 (1.70-3.53)

Simple and adjusted odds ratios (95% CI) for metabolic components associated with anxiety is presented in Table 4, and depression in Table 5. The metabolic components found to be associated with anxiety at univariate analysis included systolic BP, fasting BG, fasting BT and BMI. However, multivariable analysis revealed systolic BP (AOR = 1.009, 95% CI: 1.002 - 1.017), fasting BG (AOR = 1.002, 95% CI: 1.000 - 1.003) and fasting BT (AO R = 1.002, 95% CI: 1.001 - 1.004) to be associated with anxiety. Metabolic components found to be associated with depression at univariate analysis as well as at multivariate analysis were systolic BP (AOR = 1.017, 95% CI: 1.007 - 1.027), fasting BG (AOR = 1.004, 95% CI: 1.003 - 1.006), fasting BT (AOR = 1.004, 95% CI: 1.002 - 1.005) and BMI (AOR = 1.030, 95% CI: 1.002 - 1.058).

**Table 4:** Univariate and multivariable analysis for metabolic factors associated with anxiety among study participants

Characteristics	Crude β (SE)	p *-*value	OR (95% CI)	Adjusted β (SE)	p-value	AOR (95% CI)
Systolic BP*	0.009 (0.004)	0.015	1.009 (1.002-1.017)	0.009 (0.004)	0.013	1.009 (1.002-1.017)

Diastolic BP*	0.003 (0.006)	0.570	1.003 (0.992-1.015)	--	--	--

Fasting BG**	0.002 (0.001)	0.048	1.002 (1.000-1.003)	0.002 (0.001)	0.080	1.002 (1.000-1.003)

Fasting BT***	0.003 (0.001)	0.002	1.003 (1.001-1.004)	0.002 (0.001)	0.003	1.002 (1.001-1.004)

BMI****	0.017 (0.013)	0.195	1.017 (0.991-1.045)	--	--	--

*BP: Blood Pressure

**BG: Blood Glucose

***BT: Blood Triglycerides

****BMI: Body Mass Index

**Table 5:** Univariate and multivariable analysis for metabolic factors associated with depression among study participants

Characteristics	Crude β (SE)	p-value	OR (95% CI)	Adjusted β (SE)	p-value	AOR (95% CI)
Systolic BP*	0.014 (0.004)	< 0.001	1.014 (1.007-1.022)	0.017 (0.005)	0.001	1.017 (1.007-1.027)

Diastolic BP*	0.013 (0.006)	0.035	1.013 (1.001-1.025)	--	--	--

Fasting BG**	0.004 (0.001)	< 0.001	1.005 (1.003-1.006)	0.004 (0.001)	< 0.001	1.004 (1.003-1.006)

Fasting BT***	0.004 (0.001)	< 0.001	1.004 (1.002-1.005)	0.004 (0.001)	< 0.001	1.004 (1.002-1.005)

BMI****	0.036 (0.013)	0.007	1.036 (1.010-1.064)	0.029 (0.014)	0.033	1.030 (1.002-1.058)

*BP: Blood Pressure

**BG: Blood Glucose

***BT: Blood Triglycerides

****BMI: Body Mass Index

## Discussion

It is well documented that development of co-morbid anxiety and/or depression in people with diabetes not only leads to increased disease severity, complications, work disability, poor quality of life but is also associated with increased use of medical services and substantially higher health care costs [3,4,24,25].

A higher prevalence of anxiety and depression has been reported among people with chronic diseases [26,27] including diabetes [7,8]. It is also reported in general as well as in patient populations, that depression and other psychological problems are more prevalent in developing countries [28-30]. There are some possible explanations are reported about the high levels of anxiety and depression in developing countries compared to developed countries like higher level of gender inequities, social insecurity, lower level of education, greater level of poverty, financial difficulties and other forms of economic stressors [29,30]. In our study as well, high proportions of patients with type 2 diabetes were found positive for anxiety and depression, 58% and 44% respectively. These estimates are almost twice as high as found among people with diabetes in developed countries [7,15,16].

It is well known that being female is significantly associated with depression in general populations [29,30] and also among people with diabetes [15,16] and this study also showed a similar association. A possible explanation is that women play many gender specific roles, which exposes them to increased work demands and responsibilities. Furthermore, the social role attributed to women (passivity, dependence and emotional expression), allows them to be more emotional and extroversive in nature, in comparison to men [2,30]. Hence being female is an independent factor associated with depression.

Researchers [24,31] reported a significant association of age with depression and other psychological disorders. This study also showed increased age as an independent factor for depression. It is well reported that older patients face many challenges including isolation, more diseases and disabilities; hence making them more prone to developing psychological conditions [32]. Similarly, duration of diabetes is also associated with development of depression in this study and has been reported by other researchers as well [31,33]. Increased duration of the disease is known to significantly increase the risk of developing diabetic complications and health care expenditures [3], as a result such patients are more prone to develop psychological illnesses.

Due to an increased release of β-endorphins and brain neurotransmitters during exercise, physical activity is known to have protective physiological effects on depression and serves as a buffer against development of psychological illness [34,35]. Khuwaja et al [2] and Hong et al [36] identified that physical activity was inversely associated with the presence of anxiety depression among various groups of population. In our study also physical inactivity was found to be independently associated with anxiety.

Cardiovascular diseases have been identified as an independent factor for anxiety and depression in various studies among people with diabetes [4,17,27]. Similarly, a positive contribution of type 2 diabetes to increased rates of depression and/or anxiety disorders in patients with hypertension has been suggested [37]. In this study, having IHD and hypertension were also found to be independently associated with both anxiety and depression. These results reflect the fact that the likelihood of anxiety and depression increases with development of complications among people with diabetes.

There are inconsistent and conflicting findings regarding the relationship of MetS with anxiety and depression [38]. Some studies observe that MetS is associated with depression [18,19] while others have found no association with either entity [20]. Underlying issues in all such studies are that syndrome definitions were not standardized, and their use could either mask or reflect associations with their specific components. However, a core component of all such definitions is obesity and, among people with diabetes, obesity has been consistently identified as an independent factor associated with depression [18,20,39]. In our study also, BMI was found to be independently associated with depression, while associations of other MetS components (elevated systolic BP, fasting BG, fasting TG) were observed with both anxiety and depression. Our results therefore support the association of obesity and other MetS components with these conditions.

This study has some limitations to be noted. Being a cross-sectional study, we could not assess the temporal relationships between anxiety, depression and other diabetes related variables. Causality therefore cannot be attributed. Some missing values of fasting BG and fasting BT were imputed. Values of High Density Lipoproteins (another component of MetS) were largely missing; hence not analyzed. All the study participants were only from clinics of urban setting, so caution must be exercised in generalizing these results.

## Conclusion

Results of this multi-centre study, from one of the largest metropolitan cities of the developing world, highlight alarming findings which justify immediate recognition and action, both at clinical and public health levels; as both diabetes and anxiety/depression are major health issues to be prevented and treated. We suggest that all patients with diabetes should be assessed for these conditions so that they also can be managed so as to improve clinical outcomes. Health care providers engaged in diabetes care and treatment should be trained and updated regarding case-finding and management of these psychological conditions. Further research is needed to clarify the underlying mechanisms of associated factors and also to test interventions to reduce the risk of co-morbid anxiety and depression and their adverse outcomes.

## Competing interests

The authors declare that they have no competing interests.

## Authors' contributions

AKK conceived and designed the study and prepared the manuscript. SL and RD contributed in literature search, data cleaning and editing, and manuscript drafting and revisions. ISA managed, analyzed, and interpreted the data and provided constructive statistical feedback. GR contributed in data interpretation and provided subject feedback. FW supervised the entire project and provided intellectual feedback throughout. All authors read and approved the final manuscript.
